# In-frame editing of transcription factor gene *RDD1* to suppress miR166 recognition influences nutrient uptake, photosynthesis, and grain quality in rice

**DOI:** 10.1038/s41598-022-14768-9

**Published:** 2022-06-24

**Authors:** Masao Iwamoto

**Affiliations:** Division of Crop Genome Editing, Institute of Agrobiological Sciences, NARO, Tsukuba Ibaraki, 305-8604 Japan

**Keywords:** Plant molecular biology, Plant physiology, Molecular engineering in plants

## Abstract

The transcription factor-encoding gene *RDD1* increases the uptake of nutrient ions, photosynthetic activity under ambient and high CO_2_ conditions, and grain productivity, and microRNA166 (miR166) regulates its transcript levels. This study found that CRISPR/Cas9 genome editing of rice plants to inhibit miR166–*RDD1* transcript pairing (*R1*-Cas plants) increased *RDD1* transcript levels, NH_4_^+^ and PO_4_^3−^ uptake, and photosynthetic activity under high CO_2_ conditions in rice. However, the panicle weight of the *R1*-Cas plants decreased compared with the wild-type (WT) plants. Adversely, changes in environmental conditions, such as high CO_2_ or high temperatures, showed insignificant differences in the panicle weight between the WT and *R1*-Cas plants despite a largely increased panicle weight observed in the transgenic *RDD1*-overexpressing plants. Moreover, both the *R1*-Cas and transgenic *RDD1*-overexpressing plants that were matured in a growth chamber demonstrated an improved grain appearance quality or a decrease in the number of chalky grains compared with the WT plants. These results suggest that the in-frame mutagenesis of *RDD1* to suppress miR166–*RDD1* transcript pairing contributes to the improved grain appearance of rice.

## Introduction

There are 58 families of transcription factors in higher plants, which regulate the transcript levels of target genes^1^. DNA-binding with one finger (Dof) proteins are plant-specific transcription factors with a highly conserved 52-amino acid Dof domain at the N-terminal region. The Dof domain has a single C_2_–C_2_ zinc-finger structure that recognizes the (A/T)AAAG sequence in the promoter region of target genes^2^. Furthermore, the C-terminal region of Dof proteins contains a transcriptional activation domain^3,4^. In rice, 30 genes encoding Dof transcription factors have been identified through database searches^5^. We previously reported the characterization of a Dof transcription factor-encoding gene in rice–*rice Dof daily fluctuations 1* (*RDD1*)^6^. *RDD1* is a circadian clock-regulated gene, and daily oscillations in its expression were retained when rice plants were transferred to continuous dark or light conditions. Antisense *RDD1* transgenic plants, produced to examine the *RDD1* role, have observable reductions in grain size and weight, thus indicating that this gene is associated with rice productivity. Conversely, we found that microRNA166 (miR166) regulated the *RDD1* transcript levels, and inhibition of miR166–*RDD1* mRNA pairing was necessary for the constitutive expression of the *RDD1* transgene^7^. We previously generated transgenic rice plants overexpressing an *RDD1* transgene with nucleotide residue substitutions in the miR166 recognition site under the control of a constitutive promoter (m*RDD1*-OX plants). These transgenic rice plants experienced increased uptake and accumulation of various nutrients, including essential plant nutrients, as well as an increase in photosynthetic CO_2_ assimilation and grain productivity^7,8^. Phylogenetic analysis revealed three *RDD1*-like genes (*RDD2*, *RDD3*, and *RDD4*), which had circadian-regulated expression levels like *RDD1*^6^. However, *RDD2* function and expression patterns differed from those of *RDD1*, despite *RDD2* exhibiting the highest sequence similarity to *RDD1* among the rice Dof transcription factor genes^9^. However, *RDD4* contributed to the control of the content of various nutrient ions and the control of photosynthetic CO_2_ assimilation, as was the case with *RDD1*^10^. Additionally, it has been reported that plants overexpressing *OsDof12* (another name for *RDD4*) driven by the maize (*Zea mays*) actin promoter exhibited early flowering under long-day conditions, and its architecture was affected^11,12^. Note that the *RDD1*-like genes, except for *RDD1*, have no miR166 recognition site.

The clustered regularly interspaced short palindromic repeat (CRISPR)/CRISPR-associated endonuclease 9 (CRISPR/Cas9) system is a powerful tool for site-directed mutagenesis in various organisms, including plants. This system induces DNA double-strand breaks at given genome sites, repaired via nonhomologous end joining. However, nonhomologous end joining is error-prone. Therefore, it is commonly used to disrupt genes by generating random insertions or deletions at target sites^13^. The CRISPR/Cas9 system requires two components, Cas9 nuclease and a guide RNA (gRNA) for directing Cas9 to the target site^14^, and sequence specificity is achieved by changing a 20-nucleotide (nt) guide sequence into the gRNA. Additionally, a trinucleotide protospacer adjacent motif (PAM) is needed immediately after the target site for binding and cleavage by Cas9. In this study, we performed an in-frame mutagenesis of *RDD1* to suppress miR166–*RDD1* transcript pairing using the CRISPR/Cas9 system and examined whether endogenous *RDD1* without miR166 recognition increased its transcript levels, nutrient ion uptake, photosynthetic activity, and productivity, as observed in similar experiments involving m*RDD1*-OX plants.

## Methods

### Plant materials and growth conditions

The rice (*Oryza sativa* L. cv. Nipponbare) plants used in this study were grown in soil or in a hydroponic nutrient solution, as described previously^10^. Seedlings were maintained in a growth chamber at 28 °C under long-day conditions consisting of a 16 h light/8 h dark cycle, and white light (80 µmol·m^−2^·s^−1^) was provided using cool, white fluorescent light fixtures. Plants were grown hydroponically in a growth chamber to examine transcript levels and nutrient ion uptake. A hydroponic nutrient solution composed of 0.5 mM NH_4_H_2_PO_4_, 1 mM KNO_3_, 0.5 mM MgSO_4_, 0.5 mM CaCl_2_, 12.5 µM Fe-EDTA, and additional micronutrients (23.1 µM boron, 3.2 µM manganese, 0.1 µM copper, 1.4 µM zinc, and 0.2 µM molybdenum) was diluted 2.5 times to prepare a low-concentration hydroponic nutrient solution. To measure photosynthetic parameters and productivity, plants were transferred to growth chambers at 28 °C (light period) and 23 °C (dark period) under a 14 h light/10 h dark cycle^8^. The CO_2_ concentration in the growth chambers was set at 400 µmol mol^−1^ for plants under ambient CO_2_ conditions and 1000 µmol mol^−1^ for those under high CO_2_ conditions. The light was provided using a metal halide lamp. Examination of productivity under high-temperature conditions was conducted using plants that were initially grown in growth chambers at 28 °C (light period) and 23 °C (dark period) under a 14 h light/10 h dark cycle and then at 30 °C (light period) and 26 °C (dark period) under a 14 h light/10 h dark cycle after flowering. The collection of plant materials and the experimental research on plants were performed in accordance with relevant institutional, national, and international guidelines and legislation, and permission was obtained to collect the rice seeds.

### Construction of a gRNA/Cas9 vector and plant transformation

The construction of a gRNA/Cas9 all-in-one vector (pZH_OsU6-gRNA_MMCas9) was described^15^. Oligonucleotide pairs for the target sequence (5'-GGGATCAAGCCTGGAGACCC-3') were annealed, after which the resulting fragment was cloned into a *Bbs*I site of the gRNA cloning vector, pU6_ccdB_gRNA. This construct was digested with I-*Sce*I, and inserted into the binary vector pZH_gYSA_MMCas9. The plasmid construct was introduced into *Rhizobium radiobacter* (*Agrobacterium tumefaciens*) EHA105^16^, following which the bacteria were utilized to transform the rice plants as previously described^17^.

### RNA extraction and reverse transcription polymerase chain reaction

Plants were harvested 2 h before, or 2, 4, 6, 10, or 14 h after the onset of illumination, and total RNA was extracted using an RNeasy™ Plant Mini Kit (Qiagen, Hilden, Germany) following the manufacturer’s instructions. Reverse transcription polymerase chain reaction (RT-PCR) was conducted using one-step reactions (Superscript One-Step RT-PCR system; Invitrogen, Carlsbad, CA, USA), as previously described^6^. The primers are listed in Supplementary Table S1. The primers used for targeting *RUBQ2* were previously reported^18^. PCR was conducted in a DNA thermal cycler (GeneAmp PCR system 9700; Applied Biosystems, Foster City, CA, USA). The RT-PCR product levels were quantified using a Qubit® dsDNA HS assay kit (Invitrogen) and measured using a Qubit® 3.0 Fluorometer (Invitrogen).

### Determination of nutrient ions

The low concentration hydroponic nutrient solution was replaced with a fresh nutrient solution, and samples were collected 48 h after replacement. They were filtered using a 0.2-µm 13AI filter (GL Sciences, Tokyo, Japan). The nutrient ions in the hydroponic nutrient solution were determined by ion chromatography using the ICS-900 ion chromatography system and IonPac AS12A or CS12A column (Dionex, Sunnyvale, CA, USA).

### Photosynthesis and chlorophyll contents

The photosynthetic parameters of the upper parts of the youngest fully expanded leaf blades (no flag leaves) on the main stems at the vegetative stage, including photosynthetic carbon assimilation (*A*), stomatal conductance to water vapor (*g*_sw_), and intercellular CO_2_ concentration (*C*_i_), were determined at approximately midday using a portable CO_2_/H_2_O gas exchange analyzer (LI-6400; LI-COR, Lincoln, NE, USA), as previously described^10^. The incident photosynthetic photon flux density was 1200 µmol m^−2^ s^−1^ (light-saturated level of *A* for the rice plants), and the flow rate and leaf temperature were set to 500 µmol s^−1^ and 28 °C, respectively. CO_2_ concentrations within the leaf chambers were maintained at 400 and 1000 µmol mol^−1^ for plants grown under ambient and high CO_2_ conditions, respectively.

The chlorophyll content (soil and plant analyzer development [SPAD] values) of the leaves was examined using a chlorophyll meter (SPAD-502 Plus, Konica Minolta, Tokyo, Japan). The SPAD values were determined at the upper portion of the youngest fully expanded leaf blades on the main stems at approximately midday when the photosynthetic parameters were measured.

### Determination of glucose and starch contents

The glucose and starch contents in the rice grains were quantified using an F-kit system (Starch, R-Biopharm AG, Darmstadt, Germany) following the manufacturer’s instructions.

### Statistical analysis

Data are presented as the average ± standard error of the mean. Significant differences based on the two-sided Student’s *t*-test were indicated by asterisks (*p < 0.05, **p < 0.01, and ***p < 0.001).

## Results

### Mutagenesis of *RDD1* mediated by the CRISPR/Cas9 system

**Figure 1:**
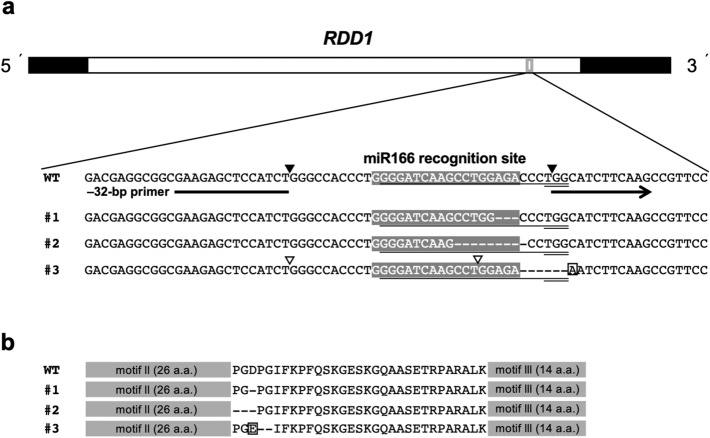
In-frame mutations around the *RDD1* miR166 recognition site. (**a**) Nucleotide sequences of *RDD1* in the wild-type (WT) and three independent lines of *R1*-Cas plants (#1–3). The untranslated and coding regions are indicated by black and white bars, respectively. The nucleotide sequences of the miR166 recognition site are boxed in gray, and the 20-nt target sequence and protospacer adjacent motif (PAM) are indicated by single and double underlines, respectively. Black and white arrowheads show the boundary sites of the 32-bp and 23-bp regions, respectively. The primer used to detect the absence of the 32-bp region (− 32-bp primer) is indicated by an interrupted arrow. A substituted nucleotide residue (adenine) in the *R1*-Cas #3 plants is shown framed by a square. (**b**) The amino acid sequences of RDD1 in the WT and *R1*-Cas #1–3 plants. Two highly conserved motifs (motifs II and III) are boxed in gray, and a substituted amino acid (glutamic acid) in the *R1*-Cas #3 plants is framed by a square.

CRISPR/Cas9 genome editing was used to generate in-frame deletions around the miR166 recognition site in *RDD1*, and three independent lines of edited rice plants (*R1*-Cas plants) were isolated (Fig. 1a). *R1*-Cas #1 and #2 plants revealed 3- and 9-bp deletions, respectively, including the miR166 recognition site, and *R1*-Cas #3 plants carried a 6-bp deletion and substitution of 1 nt in the adjacent downstream region of the miR166 recognition site. The deleted amino acid residues in all *R1*-Cas plant lines were located between motifs II and III, both of which encode highly conserved amino acid sequences in *RDD1*-like genes in plants^6^ (Fig. 1b). It was reported that five Class III homeodomain–leucine zipper (Class III HD–Zip) genes (*OSHB1*, *OSHB2*, *OSHB3*, *OSHB4*, and *OSHB5*) in rice have the miR166 recognition site, and they carry the same conserved recognition site sequence^19^. However, a comparison of the 20-nt target sequence of *RDD1* with corresponding sequences of Class III HD–Zip and *RDD1-*like genes showed no sequence homology > 70% (Supplementary Fig. S1).

### Effects of *RDD1* mutations on *RDD1* expression and miR166 recognition

**Figure 2:**
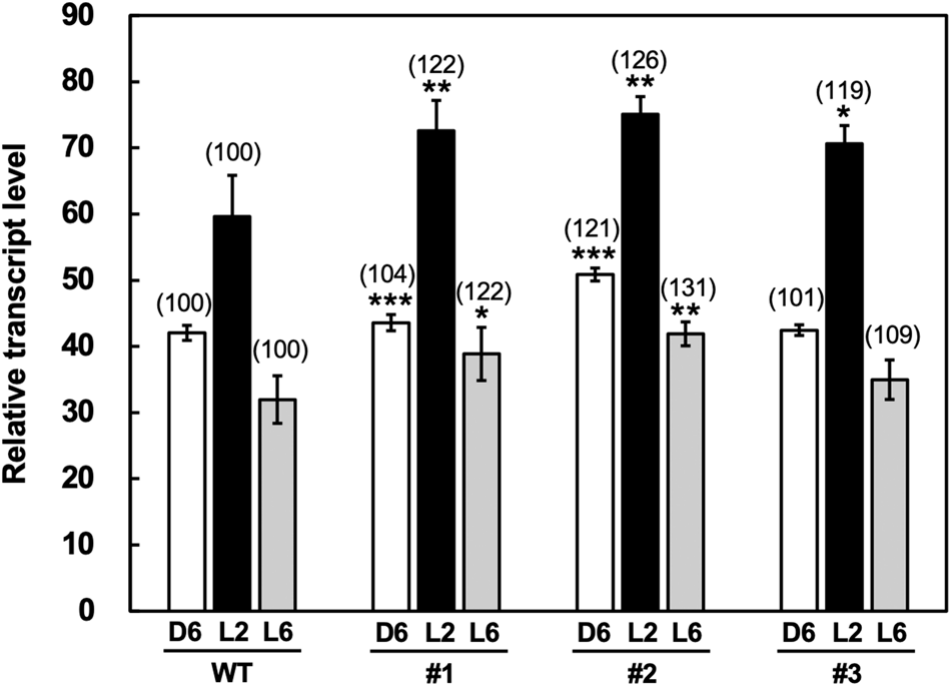
*RDD1* transcript levels in the shoots of the wild-type (WT) and *R1*-Cas #1–3 plants at the 4-leaf stage. The plants were grown in a low concentration hydroponic nutrient solution under normal conditions and harvested 2 h before (D6), and 2 h (L2), and 6 h (L6) after the onset of illumination. The transcript levels are normalized to those of *RUBQ2*, and the highest transcript level is defined as 100. Data are presented as the average ± standard error of the mean (*n* = 8). Asterisks indicate significant differences relative to the WT plants based on the Student’s *t*-test (*p < 0.05, **p < 0.01, and ***p < 0.001). At the time of each harvest, the values relative to those of the WT as 100 are shown in parentheses.

The *RDD1* transcript levels oscillate daily, peaking after dawn under long-day conditions^6^. Examination of the *RDD1* transcript levels in the *R1*-Cas plants indicated that the *RDD1* transcript levels in the peak (L2) increased 1.2- to 1.3-fold in the *R1*-Cas plants compared with the wild-type (WT) plants (Fig. 2). A decline in the *RDD1* transcript levels before and after the peak (D6 and L6, respectively) was also detected in the *R1*-Cas and WT plants, although the *RDD1* transcript levels in the *R1*-Cas #1 and #2 plants increased compared with the levels in the WT plants at D6 and L6.

**Figure 3:**
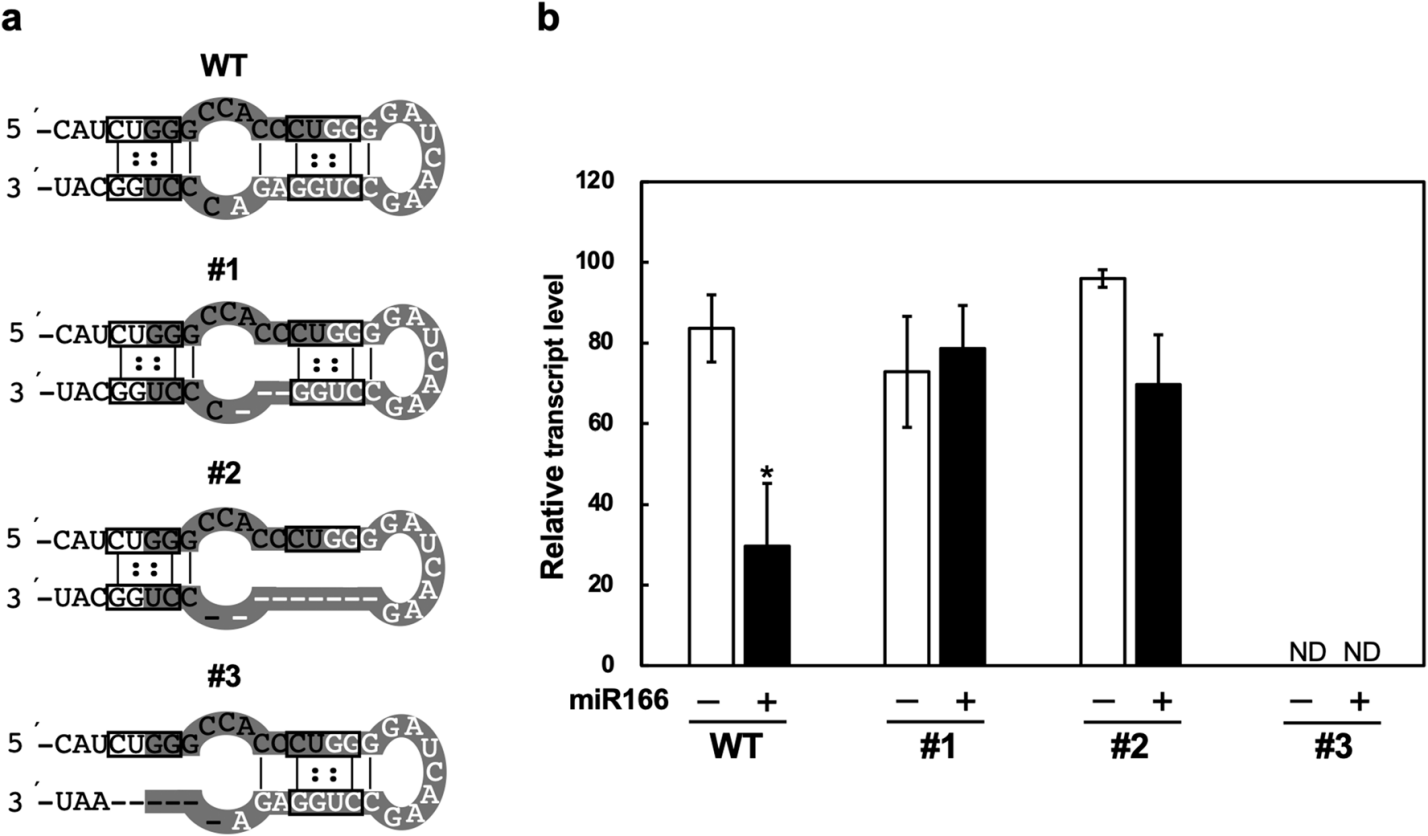
Effects of *RDD1* mutations on the deletion of the 32-bp region from the RT-PCR products by adding synthetic miR166. (**a**) Inferred secondary structures of the 32-bp region in the *RDD1* transcripts of the wild-type (WT) and *R1*-Cas #1–3 plants. Nucleotide sequences corresponding to the 32-bp region are shaded, and those of the miR166 recognition site are denoted by white letters. 5'-CUGG-3' sequences are boxed and G–U base pairs are denoted by double dots. (**b**) RT-PCR amplification of *RDD1* in addition to synthetic miR166. Total RNAs from the shoots of the WT and *R1*-Cas #1–3 plants at the 4-leaf stage were reverse-transcribed in the absence (−) or presence (+) of synthetic miR166 RNA. The plants were grown in a low concentration hydroponic nutrient solution under normal conditions and harvested 2 h after the onset of illumination. The transcript levels are normalized to those of *RUBQ2*, and the highest transcript level is defined as 100. Data are presented as the average ± standard error of the mean (*n* = 3). An asterisk indicates a significant difference relative to the transcript level in the absence of synthetic miR166 based on the Student’s *t*-test (*p < 0.05). ND, RT-PCR products could not be amplified due to a partial deletion of the annealing site for the − 32-bp primer (see Fig. 1a).

The full-length cDNA for *RDD1* (DDBJ/EMBL/GenBank database accession number AK063318) lacks a 32-bp region in the coding region. Our previous study reported that the miR166 recognition site is located within the 32-bp region, which deletes artificially during a reverse transcription reaction in RT-PCR analysis, and that adding synthetic miR166 RNA strongly inhibited cDNA synthesis of the *RDD1* transcript lacking the 32-bp region^7^. The 32-bp region appears to form a stem-loop structure, an architecture that might cause the 32-bp region’s deletion during reverse transcription. The inferred stem-loop structure is possible in the *R1*-Cas #1 and #2 plants but not in the *R1*-Cas #3 plants (Fig. 3a). RT-PCR analysis was conducted to ascertain miR166 RNA binding to the miR166 recognition site in the *R1*-Cas plants. We found that RT-PCR equally amplified the RT-PCR product lacking the nucleotide sequence corresponding to the 32-bp region with or without synthetic miR166 RNA in the *R1*-Cas #1 and #2 plants, although adding synthetic miR166 RNA inhibited the amplification of the RT-PCR product lacking the 32-bp region in the WT plants (Fig. 3b). These results showed that the binding of synthetic miR166 RNA to the miR166 recognition site was suppressed in the *R1*-Cas #1 and #2 plants. Conversely, there was no amplification of the RT-PCR product in the *R1*-Cas #3 plants because of a 2-bp deletion within the − 32-bp primer annealing site (Fig. 1a). Although the *R1*-Cas #3 plants lost the 3'-boundary sequence of the 32-bp region, an identical nucleotide sequence within the miR166 recognition site could pair with the 5'-boundary sequence of the 32-bp region, forming a 23-bp stem-loop structure (Fig. 1a and Supplementary Fig. S2a,b). We examined whether the inferred 23-bp stem-loop structure was formed in the *R1*-Cas #3 plants. The RT-PCR analysis indicated that the RT-PCR product lacking the 23-bp region was equally amplified, irrespective of the addition of synthetic miR166 RNA during reverse transcription (Supplementary Fig. S2c), suggesting that there was no synthetic miR166 RNA binding to the miR166 recognition site in the *R1*-Cas #3 plants.

### Nutrient ion uptake in *R1*-Cas plants

**Figure 4:**
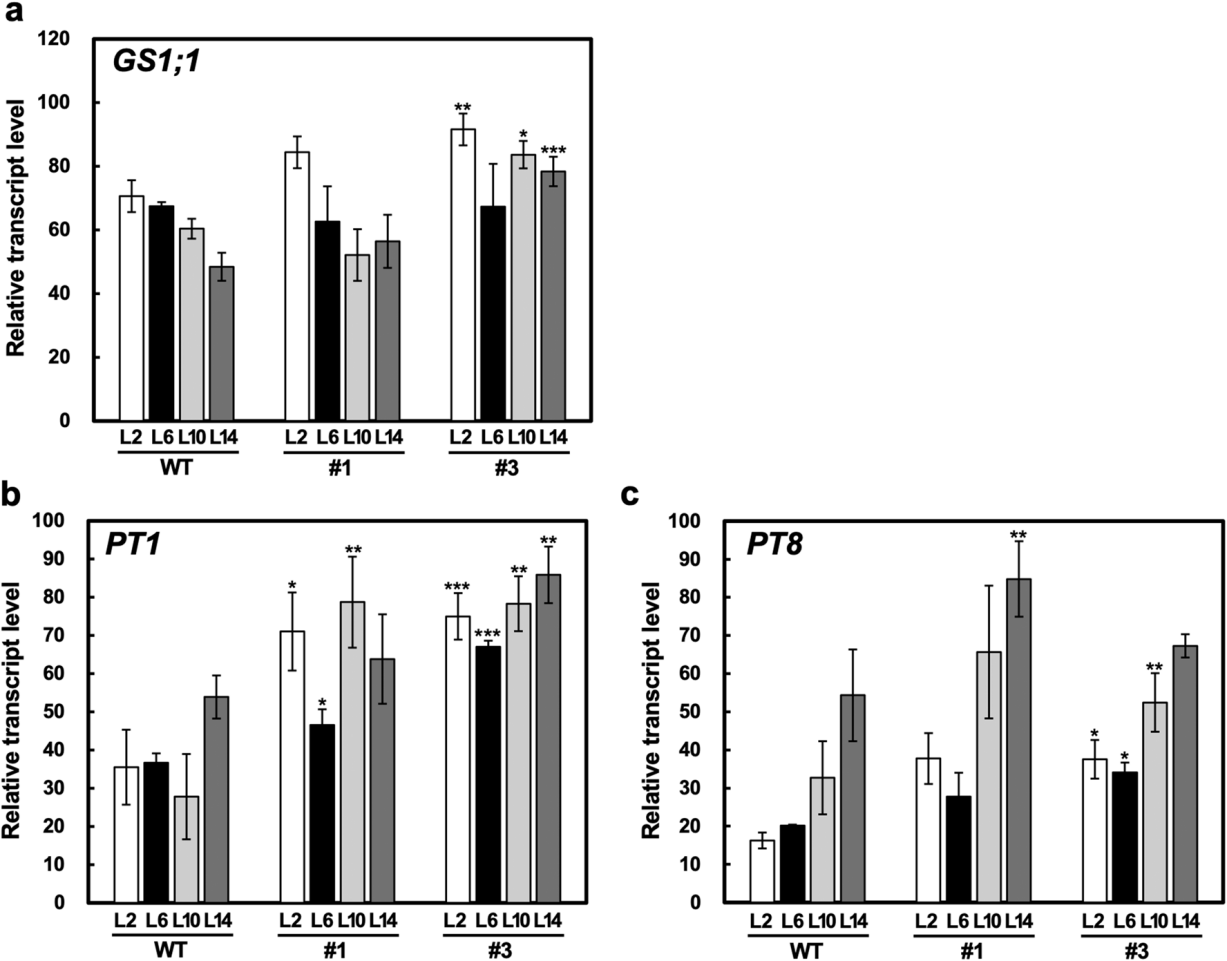
Transcript levels of *GS1;1* (**a**), *PT1* (**b**), and *PT8* (**c**) in the shoots of the wild-type (WT) and *R1*-Cas #1 and #3 plants at the 4-leaf stage. The plants were grown in a low concentration hydroponic nutrient solution under normal conditions and harvested 2 h (L2), 6 h (L6), 10 h (L10), and 14 h (L14) after the onset of illumination. The transcript levels are normalized to those of *RUBQ2*, and the highest transcript level is defined as 100. Data are presented as the average ± standard error of the mean (*n* = 3). Asterisks indicate significant differences relative to the WT plants based on the Student’s *t*-test (*p < 0.05, **p < 0.01, and ***p < 0.001).

Next, we examined the nutrient ion uptake in the *R1*-Cas plants under low-nutrient conditions as m*RDD1*-OX plants have been reported to increase the absorption of nutrient ions under low-nutrient conditions^7^. The nutrient ion uptake was examined by comparing the nutrient ion content in the hydroponic nutrient solution at 48 h after replacement with the fresh hydroponic nutrient solution containing the initial nutrient ion content. The absorption of the nutrient ions, except for SO_4_^2−^ and Cl^−^, per shoot fresh weight (FW) was greater in two of the *R1*-Cas plant lines than in the WT plants (Table 1). However, the shoot FW of the *R1*-Cas plants was significantly lower than that of the WT plants grown in the hydroponic nutrient solution (Supplementary Table S2). Also, the values of NH_4_^+^ and NO_3_^−^ uptake in the *R1*-Cas plants relative to the WT plants in Table 1 were identical to those of the shoot FW of the WT plants relative to the *R1*-Cas plants in Supplementary Table S2, indicating that most of the NH_4_^+^ and NO_3_^−^ in the hydroponic nutrient solution were absorbed in both the WT and *R1*-Cas plants. Alternatively, when the remaining nutrient ion content in the hydroponic nutrient solution at 48 h after replacement was compared between the WT and *R1*-Cas plants, the NH_4_^+^ and PO_4_^3−^ content in the hydroponic nutrient solution was significantly lower in the *R1*-Cas plants than in the WT plants, despite the lower shoot FW in the *R1*-Cas plants (Table 1). Our previous study showed that *RDD1* overexpression induced the expression of genes for glutamine synthetase 1;1 (GS1;1) and phosphate transporters 1 and 8 (PT1 and PT8, respectively), which are associated with the transport of NH_4_^+^ and PO_4_^3−^, respectively^7^. Therefore, transcript levels of *GS1;1*, *PT1*, and *PT8* were examined in *R1*-Cas plants, and it was found that except for the transcript level of *GS1;1* in the *R1*-Cas #1 plants, all transcript levels increased in the *R1*-Cas plants (Fig. 4).

**Table 1:** Nutrient ion uptake and remaining nutrient ion content in the hydroponic nutrient solution at 48 h after replacement with the fresh hydroponic nutrient solution in the WT, and *R1*-Cas #1 as well as #3 plants at the 5-leaf stage.

		µ mol g^−1^ FW	µ mol
		Uptake (48 h)	Content in hydroponic nutrient solution (48 h)
NH_4_^+^	WT	26.626 ± 0.010 (100)	0.144 ± 0.025 (100)
	#1	34.724 ± 0.004*** (130)	0.051 ± 0.007* (35)
	#3	35.477 ± 0.001*** (133)	0.013 ± 0.003** (9)
NO_3_^−^	WT	60.385 ± 0.002 (100)	0.019 ± 0.006 (100)
	#1	78.643 ± 0.001*** (130)	0.015 ± 0.001 (75)
	#3	80.301 ± 0.002*** (133)	0.018 ± 0.004 (96)
PO_4_^3−^	WT	15.174 ± 0.072 (100)	41.370 ± 0.182 (100)
	#1	21.155 ± 0.083*** (139)	38.674 ± 0.160*** (93)
	#3	23.589 ± 0.031*** (155)	34.908 ± 0.059*** (84)
K^+^	WT	57.697 ± 0.045 (100)	26.763 ± 0.114 (100)
	#1	69.096 ± 0.013*** (120)	38.459 ± 0.025*** (144)
	#3	62.271 ± 0.035*** (108)	54.156 ± 0.066*** (202)
Mg^+^	WT	9.346 ± 0.102 (100)	48.484 ± 0.258 (100)
	#1	12.889 ± 0.017*** (138)	47.095 ± 0.032* (97)
	#3	11.052 ± 0.008*** (118)	51.092 ± 0.015** (105)
SO_4_^2−^	WT	8.799 ± 0.060 (100)	48.461 ± 0.152 (100)
	#1	9.012 ± 0.087** (102)	53.196 ± 0.168*** (110)
	#3	7.377 ± 0.067*** (84)	56.654 ± 0.128*** (117)
Ca^2+^	WT	5.077 ± 0.114 (100)	54.583 ± 0.288 (100)
	#1	6.635 ± 0.023*** (131)	54.539 ± 0.044 (100)
	#3	5.621 ± 0.007* (111)	56.725 ± 0.013** (104)
Cl^−^	WT	39.493 ± 0.046 (100)	50.756 ± 0.115 (100)
	#1	45.672 ± 0.061*** (116)	61.903 ± 0.118*** (121)
	#3	29.346 ± 0.082*** (74)	94.667 ± 0.155*** (187)

Data are represented as the average ± standard error of the mean (*n* = 4). Asterisks indicate significant differences relative to the WT plants based on the Student’s *t*-test (*P < 0.05, **P < 0.01 and ***P < 0.001). Values relative to those of WT as 100 are shown in parentheses.

### Photosynthetic activity in *R1*-Cas plants

**Figure 5:**
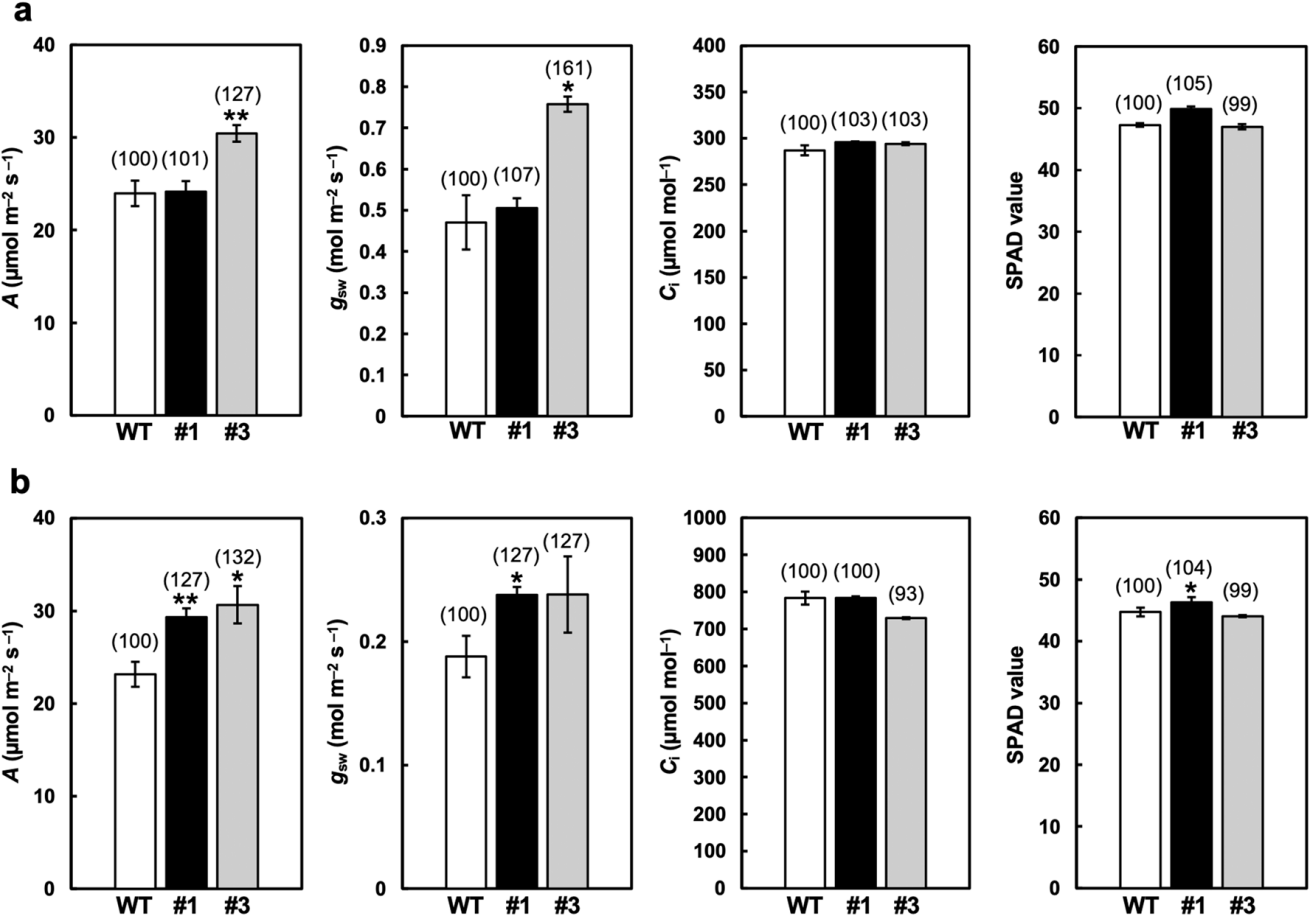
Photosynthesis-related characteristics in the *R1*-Cas plants. The photosynthetic carbon assimilation (*A*), stomatal conductance to water vapor (*g*_*sw*_), intercellular CO_2_ concentration (*C*i), and SPAD value of the wild-type (WT) and *R1*-Cas #1 and #3 plants in a growth chamber under ambient CO_2_ (**a**) and high CO_2_ conditions (1000 µmol CO_2_ mol^-1^) (**b**). Data are presented as the average ± standard error of the mean (*n* = 3). Asterisks indicate significant differences relative to the WT plants based on the Student’s *t*-test (*p < 0.05 and **p < 0.01). Values relative to those of WT as 100 are shown in parentheses.

The *R1*-Cas plants’ photosynthetic activity was examined to determine whether an in-frame deletion of *RDD1* to suppress the miR166 recognition promoted the photosynthetic activity. The photosynthetic CO_2_ assimilation rates of the *R1*-Cas #1 and #3 plants exhibited no difference and a 1.3-fold increase, respectively, compared with those of the WT plants grown under ambient CO_2_ conditions (400 µmol CO_2_ mol air^−1^) (Fig. 5a). Stomatal conductance was higher in the *R1*-Cas #3 plants than in the WT plants, and there were insignificant differences in intercellular CO_2_ concentrations in either the *R1*-Cas #1 or #3 plants. Conversely, when plants were grown under high CO_2_ conditions (1000 µmol CO_2_ mol air^-1^), we found that the photosynthetic CO_2_ assimilation rates of the *R1*-Cas #1 and #3 plants were 1.3-fold higher than those of the WT plants (Fig. 5b). Additionally, we examined the chlorophyll content (SPAD value) of the plants and observed that there was no significant difference in the SPAD value between the WT and *R1*-Cas plants under both ambient and high-CO_2_ conditions. However, the SPAD value of the *R1*-Cas #1 plants was statistically higher than that of the WT plants under high CO_2_ conditions.

### Effects of *RDD1* mutation on productivity

The increased photosynthetic CO_2_ assimilation rates of the *R1*-Cas plants under high CO_2_ conditions suggested that productivity may be higher in the *R1*-Cas plants than in the WT plants under high CO_2_ conditions. The *R1*-Cas plants were matured in a growth chamber grown under both ambient and high CO_2_ conditions to examine their productivity. No significant difference was found in the panicle dry weight (DW) between the WT and *R1*-Cas plants under high CO_2_ conditions despite a decreased panicle DW in the *R1*-Cas plants under ambient CO_2_ conditions (Table 2). Particularly, the panicle DW in the m*RDD1*-OX plants was 1.4-fold higher than that in the WT plants under high CO_2_ conditions. Conversely, the shoot DW in the *R1*-Cas plants was lower than that in the WT plants under both ambient and high CO_2_ conditions, as observed in the m*RDD1*-OX plants. Consequently, the panicle DW to shoot DW ratio was significantly higher in the *R1*-Cas plants than in the WT plants under high CO_2_ conditions but not under ambient CO_2_ conditions. Note that the m*RDD1*-OX plants showed the highest panicle DW to shoot DW ratio under both ambient and high CO_2_ conditions.

**Table 2:** Characterization of shoots and panicles of mature WT, *R1*-Cas #1 and #3, and m*RDD1*-OX plants (m*R1*-OX) at the mature stage under ambient and high CO_2_ conditions.

	CO_2_	WT	#1	#3	m*R1*-OX
Shoot DW	Ambient	54.102 ± 2.179 (100)	37.940 ± 3.626** (70)	37.846 ± 3.770** (70)	39.492 ± 2.934** (73)
	High	74.183 ± 3.908 (100)	56.108 ± 9.746 (76)	56.788 ± 6.803 (77)	57.229 ± 0.609* (77)
Panicle DW	Ambient	10.271 ± 0.371 (100)	7.983 ± 1.505 (78)	7.625 ± 1.003* (74)	11.905 ± 1.219 (116)
	High	15.503 ± 0.562 (100)	15.397 ± 2.671 (99)	14.067 ± 1.647 (91)	21.010 ± 0.375** (136)
Panicle DW/Shoot DW ratio	Ambient	0.191 ± 0.009 (100)	0.217 ± 0.040 (114)	0.207 ± 0.030 (109)	0.301 ± 0.016** (158)
	High	0.209 ± 0.006 (100)	0.274 ± 0.004** (131)	0.265 ± 0.012* (127)	0.377 ± 0.006*** (180)

Data are represented as the average ± standard error of the mean (*n* = 3–4). Asterisks indicate significant differences relative to the WT plants based on the Student’s *t*-test (*P < 0.05, **P < 0.01 and ***P < 0.001). Values relative to those of WT as 100 are shown in parentheses. DW, dry weight.

High temperature is a major serious environmental problem caused by global climate change. High temperatures occurring during rice ripening periods decrease grain yield and quality. To examine whether the *R1*-Cas plants exhibited improved productivity under high-temperature stress, the *R1*-Cas plants were initially grown in a growth chamber under normal temperature and transferred to high-temperature conditions after flowering. No statistically significant difference was found in the panicle DW between the WT and *R1*-Cas plants, although the panicle DW was 2.4-fold higher in the m*RDD1*-OX plants than in the WT plants (Table 3). Conversely, the shoot DW was significantly decreased in the *R1*-Cas plants compared with that in the WT plants under high-temperature conditions, as observed in the m*RDD1*-OX plants. Therefore, the panicle DW to shoot DW ratio in the m*RDD1*-OX plants was significantly higher than that in the WT plants. Because of high-temperature stress, the one-grain weight decreased in all plants examined. Nevertheless, the one-grain weight was 1.2-fold higher in the m*RDD1*-OX plants than in the WT plants under high-temperature conditions.

**Table 3:** Characterization of shoots, panicles, and grains of mature WT, *R1*-Cas #1 and #2, and m*RDD1*-OX plants (m*R1*-OX) at the mature stage under high-temperature conditions.

	WT	#1	#2	m*R1*-OX
Shoot DW (g)	72.573 ± 1.038 (100)	36.334 ± 2.359** (50)	38.033 ± 0.654*** (52)	41.889 ± 4.410* (58)
Panicle DW (g)	3.770 ± 0.497 (100)	1.992 ± 0.306 (53)	4.100 ± 1.016 (109)	9.151 ± 0.566* (243)
Panicle DW/Shoot DW ratio	0.052 ± 0.007 (100)	0.054 ± 0.005 (104)	0.108 ± 0.027 (207)	0.221 ± 0.017* (425)
One-grain DW (mg)	14.214 ± 0.251 (100)	14.050 ± 0.232 (99)	15.179 ± 0.422 (107)	17.052 ± 0.050** (120)

Data are represented as the average ± standard error of the mean (*n* = 3). Asterisks indicate significant differences relative to the WT plants based on the Student’s *t*-test (*P < 0.05, **P < 0.01 and ***P < 0.001). Values relative to those of WT as 100 are shown in parentheses. DW, dry weight.

### Grain appearance quality in *R1*-Cas plants

**Figure 6:**
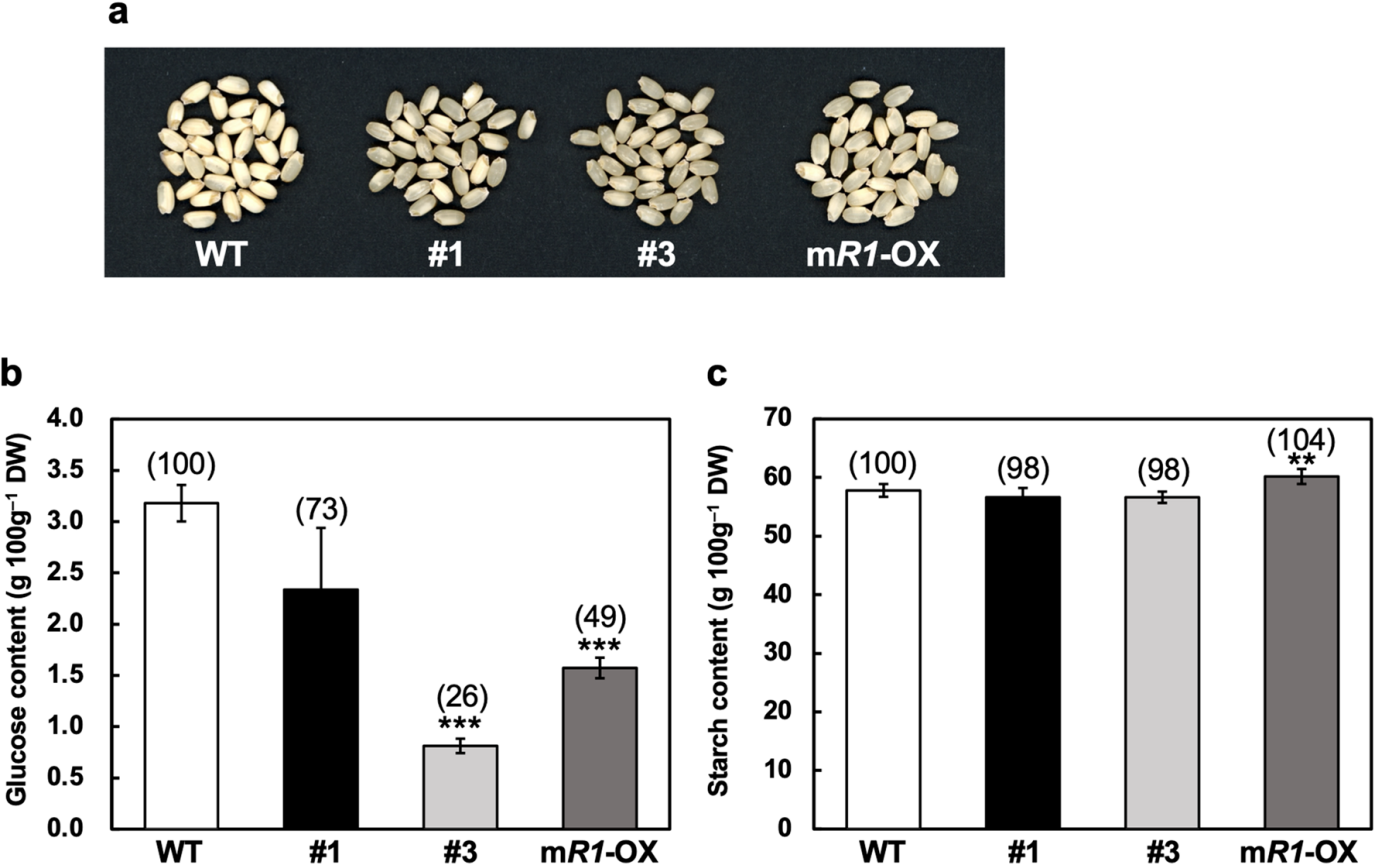
Differences in the rice grains’ chalky appearance and glucose as well as starch contents of plants grown in a growth chamber under normal conditions. (**a**) Rice grains of the wild-type (WT), *R1*-Cas #1 and #3, and m*RDD1*-OX plants (m*R1*-OX). (**b**,**c**) Glucose (**b**) and starch contents (**c**) of the rice grains of the WT, *R1*-Cas #1 and #3, and m*RDD1*-OX plants (m*R1*-OX). Data are presented as the average ± standard error of the mean (*n* = 5). Asterisks indicate significant differences relative to the WT plants based on the Student’s *t*-test (**p < 0.01 and ***p < 0.001). Values relative to those of WT as 100 are shown in parentheses.

Chalky appearance is an important quality characteristic of rice grains. Chalkiness is the opaque part of the endosperm and is colored white compared to the relatively transparent rest part. When the WT and *R1*-Cas plants matured in a growth chamber under normal conditions, most of the WT plants’ rice grains appeared chalky (Supplementary Table S3 and Fig. 6a). Contrarily, the number of chalky grains in both the *R1*-Cas and m*RDD1*-OX plants was smaller than that of chalky grains observed in the WT plants. Other studies have reported that the glucose content in the opaque parts of chalky grains is remarkably high compared with that in the translucent parts of perfect grains^20^. Because of these findings, we measured the glucose content in the whole grains of the *R1*-Cas and m*RDD1*-OX plants and found that the glucose content was less in both the *R1*-Cas and m*RDD1*-OX plants than that in the WT plants (Fig. 6b). Regarding the starch content, no significant difference was found between the WT and *R1*-Cas plants, and it was slightly higher in the m*RDD1*-OX plants than in the WT plants (Fig. 6c).

## Discussion

miRNAs are small, non-coding RNAs that regulate gene expression by binding to partially complementary sequences in target mRNAs^21^. Research has shown that miRNAs affect the function of circadian clocks in flies and mammals^22^. Our previous studies demonstrated that *RDD1* overexpression driven by a constitutive promoter exhibited diurnal fluctuations through the miR166 recognition site^7^. In this study, in-frame editing of *RDD1* was conducted to increase the transcript levels of *RDD1* by interfering with miR166’s binding to *RDD1*’s recognition site. We found that the mutated *RDD1* transcript levels driven by the endogenous *RDD1* promoter were oscillated and increased 1.2- to 1.3-fold at the peak in the *R1*-Cas plants (Fig. 2). Conversely, the *RDD1* transgene was constitutively expressed under the control of a constitutive promoter in the m*RDD1*-OX plants. Because oscillations in the transcript levels of mutated *RDD1* were similar to those of the transcript levels of endogenous *RDD1* in the WT plants, we suspect that the endogenous *RDD1* promoter drives the oscillated expression in cooperation with miR166. Additionally, the RT-PCR analysis in the *R1*-Cas #3 plants suggested that synthetic miR166 did not bind to the *RDD1* transcript’s miR166 recognition site, which carries no deletion (Supplementary Fig. S2). These results suggested that the inferred 32-bp stem-loop structure is important for miR166’s pairing to the *RDD1* transcript.

Enhanced CO_2_ levels have been reported to stimulate photosynthesis by promoting the net rate of carboxylation catalyzed by ribulose-1,5-bisphosphate carboxylase/oxygenase (Rubisco)^23^. This study showed that the photosynthetic CO_2_ assimilation rates of the *R1*-Cas plants were higher than those of the WT plants under high CO_2_ conditions (Fig. 5). Our previous study reported that *RDD1* functions to promote photosynthetic activity^8^, and we found that the *RDD1* transcript levels in the wild-type plants grown under high CO_2_ conditions were significantly decreased compared with those under ambient CO_2_ conditions (Supplementary Fig. S3). These results indicated that the ability to increase the *RDD1* transcript levels by inhibiting miR166–*RDD1* transcript pairing in the *R1*-Cas plants appears to positively contribute to photosynthetic CO_2_ assimilation-related processes under high CO_2_ conditions rather than those under ambient CO_2_ conditions. Because the NH_4_^+^ and PO_4_^3−^ contents in the hydroponic nutrient solution in the *R1*-Cas #3 plants were lower than those in the *R1*-Cas #1 plants (Table 1), the NH_4_^+^ and PO_4_^3−^ uptake in the *R1*-Cas #3 plants was greater than that in the *R1*-Cas #1 plants under normal conditions. Therefore, an increased NH_4_^+^ and PO_4_^3−^ uptake in the *R1*-Cas #3 plants appears to support an increase in photosynthetic CO_2_ assimilation rates under ambient CO_2_ conditions (Fig. 5).

Protein phosphorylation is an ubiquitous mechanism for the temporal and spatial regulation of proteins, and most eukaryotic protein phosphorylation occurs at serine, threonine, and tyrosine residues^24^. Aspartic acid (Asp) and glutamic acid (Glu) are negatively charged amino acids that can sometimes mimic the phosphorylation state of a protein^25^. As shown in Fig. 1b, the *R1*-Cas #1 and #2 plants had a deleted Asp residue, whereas the *R1*-Cas #3 plants had a substitution of an Asp with a Glu residue. Therefore, the absence of Asp may have negatively influenced RDD1’s activity in the *R1*-Cas plants, and the presence of Glu instead of Asp possibly caused higher NH_4_^+^ and PO_4_^3−^ uptake, *GS1;1* and *PT1* expression, and photosynthetic CO_2_ assimilation rates under ambient CO_2_ conditions in the *R1*-Cas #3 plants than was observed in the *R1*-Cas #1 plants.

An examination of the *R1*-Cas plants’ productivity indicated that the panicle weight decreased in the *R1*-Cas plants grown under ambient CO_2_ conditions (Table 2). Therefore, the amino acid residues, which were deleted from RDD1 in the *R1*-Cas plants, may have been necessary to increase grain productivity. In-frame deletion lines of *early heading date 1* (*Ehd1*), which acts as a key signal integrator in the networks that regulate floral transition^26^, lacked one to three amino acid residues in the receiver domain using the CRISPR/Cas9 system, and their flowering time was delayed compared with that of the WT plants but earlier than that of the frame-shift *ehd1* lines^27^. These reports suggested that one to three amino acid deletions in *RDD1* yield a moderate decrease in grain productivity. Because the suppression of miR168, which targets *Argonaute1* (*AGO1*), by a target mimic improves grain yield in rice^28^, transgenic plants expressing the *RDD1* target mimic, including the 32-bp region, may show increased panicle weight via the enhanced transcript levels of endogenous *RDD1*.

High-temperature stress reduces growth, yield, and grain quality^29,30^. The *R1*-Cas plants were grown under high-temperature conditions after flowering to ascertain the effect of high temperatures on productivity, and it revealed that there was no significant difference in the panicle and one-grain weights between the WT and *R1*-Cas plants (Table 3). This result indicated that in-frame editing of *RDD1* had no significant effect on productivity under high-temperature stress. Interestingly, panicle and one-grain weights largely increased in the m*RDD1*-OX plants compared with the WT and *R1*-Cas plants under high-temperature conditions. These results suggested that constitutive *RDD1* overexpression in the m*RDD1*-OX plants, but not fluctuations of its expression in the *R1*-Cas plants, can strengthen grain yield under high-temperature stress.

The number of chalky grains in rice can be increased by high-temperature stress during grain filling^31^. The chalky appearance results from the air spaces between the loosely packed starch granules that randomly reflect light^32^. We found that the number of chalky grains in both the *R1*-Cas and m*RDD1*-OX plants significantly decreased compared with that in the WT plants when the plants matured in a growth chamber under normal conditions (Supplementary Table S3 and Fig. 6a). This indicated that in-frame mutations of *RDD1* in the *R1*-Cas plants decreased the number of chalky grains, just like the constitutive *RDD1* expression in the m*RDD1*-OX plants. Due to an increase in temperature (up to 35 °C) for a moment in a growth chamber after transferring from the dark period (23 °C) to the light period (28 °C), this may generate many chalky grains in the WT plants. Because it has been suggested that unusual starch degradation occurs in chalky grains of rice^20^, *RDD1* may negatively affect starch degradation of grains, thus decreasing the number of chalky grains. This is supported by the results revealing that the glucose content in the grains decreased in the *R1*-Cas and m*RDD1*-OX plants compared with the WT plants (Fig. 6b). Since Asp and Glu can mimic the phosphorylation state of a protein^25^, the presence of a Glu instead of an Asp residue in the *R1*-Cas #3 plants may decrease the glucose content in grains compared with the *R1*-Cas #1 plants, which had the RDD1 protein without an Asp residue.

In conclusion, this study demonstrated that in-frame editing of *RDD1* to suppress miR166’s binding to the *RDD1* transcript promoted the uptake of NH_4_^+^ and PO_4_^3−^, the expression of NH_4_^+^ and PO_4_^3−^ transport-associated genes, and grain appearance quality. Still, the panicle weight decreased in the *R1*-Cas plants. Therefore, we expect that editing *RDD1* to substitute nucleotide residue(s) but not amino acid(s) within the 32-bp region, including the miR166 recognition site, largely improves rice grain yield.

## Supplementary Information

Supplementary Information.
