# Supplementary material for: In-frame editing of transcription factor gene RDD1 to suppress miR166 recognition influences nutrient uptake, photosynthesis, and grain quality in rice
Source: Sci Rep. 2022 Jun 24;12:10795. doi: 10.1038/s41598-022-14768-9 (PMC9232572; doi:10.1038/s41598-022-14768-9)
Supplement: Supplementary file 1 — Supplementary Information. [file 41598_2022_14768_MOESM1_ESM.pdf]

Supplementary Information

In-frame editing of transcription factor gene *RDD1* to suppress miR166 recognition influences nutrient uptake, photosynthesis, and grain quality in rice

Masao Iwamoto

Supplementary Table S1. Oligonucleotide sequences of primers used in this study

Gene	Primer	Sequence (5′–3′)
RDD1	RDD1-F	CTGAAGGAGGACAAGGAGGAG
	RDD1-R	TCTCATGAAAGCCCTTTGCAACTC
RDD1 lacking 32-bp region	–32-bp-F	GAAGAGCTCCATCTGGCATCTTCAAG
	RDD1-R	TCTCATGAAAGCCCTTTGCAACTC
RDD1 lacking 23-bp region in *R1*-Cas #3 plants	#3 –23-bp-F	GAAGAGCTCCATCTGGAGAAATCTTC
	RDD1-R	TCTCATGAAAGCCCTTTGCAACTC
RDD1 containing 23-bp region in *R1*-Cas #3 plants	#3 +23-bp-F	CTGGGGATCAAGCCTGGAGAA
	RDD1-R	TCTCATGAAAGCCCTTTGCAACTC
GS1;1	GS1;1-F	GACGAGGATGTCGTGGCGTCAAGAAG
	GS1;1-R	GATGGTGGCTACGAGATCATCAAG
PT1	PT1-F	CAAGAACGCGCTCTTCGTGCTC
	PT1-R	CAACCACTCCCTGCGTGATCAC
PT8	PT8-F	CTCGGGTTCATCTGCACGTTC
	PT8-R	CTACACTATATTCACGTGAGTC

Supplementary Table S2. Shoot FW of the WT, and *R1*-Cas #1 and #3 plants at the 5-leaf stage grown in the hydroponic nutrient solution

	Shoot FW (g)
WT	0.906 ± 0.014
#1	0.643 ± 0.011*** (130)
#3	0.665 ± 0.022*** (133)

Data are represented as the average ± standard error of the mean ($n = 6$). Asterisks indicate significant differences relative to the WT plants based on the Student's *t*-test (***$P < 0.001$). Values of WT relative to those of *R1*-Cas #1 or #3 as 100 are shown in parentheses.

Supplementary Table S3. Chalky grain number of 50 brown rice grains in the WT, *R1*-Cas #1 and #3, and m*RDD1*-OX plants (m*R1*-OX) grown in a growth chamber under normal conditions

	Chalky grain
WT	45.75 ± 0.75 (91.5%)
#1	25.75 ± 2.95* (51.5%)
#3	26.00 ± 2.38** (52.0%)
m*R1*-OX	24.50 ± 3.28** (49.0%)

Data are represented as the average ± standard error of the mean (n = 4). Asterisks indicate significant differences relative to the WT plants based on the Student's *t*-test (*$P < 0.05$ and **$P < 0.01$). Percentages of chalky rice grains are shown in parentheses.

RDD1	GGGATCAAGCCTGGAGACCC	
RDD2	GGCATTGAACCTGGTGACCG	70%
RDD3	GCCTTCAGAGGCGGCGACAA	45%
RDD4	GGGATCAAGGGTGACAAGGT	60%
OSHB1	GGGATGAAGCCTGGTCCGGA	65%
OSHB2	GGGATGAAGCCTGGTCCGGA	65%
OSHB3	GGGATGAAGCCTGGTCCGGA	65%
OSHB4	GGGATGAAGCCTGGTCCGGA	65%
OSHB5	GGGATGAAGCCTGGTCCGGA	65%

Supplementary Figure S1. The alignment of nucleotide sequences corresponding to the target sequence of *RDD1*. The nucleotides, which are the same as those of *RDD1*, are boxed in gray. Sequence homologies to *RDD1* (%) are shown.

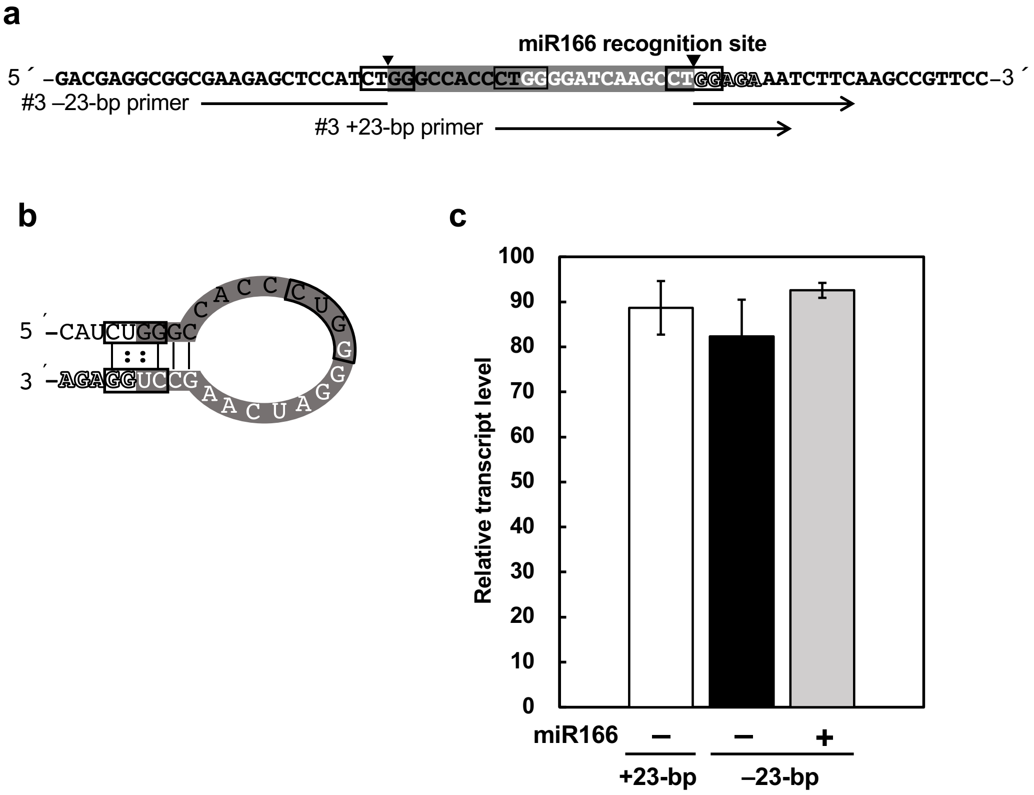

Supplementary Figure S2. Effects of an *RDD1* mutation in the *R1*-Cas #3 plants on the deletion of the predicted 23-bp region from the RT-PCR products by adding synthetic miR166. (**a**) Nucleotide sequences of edited *RDD1* in the *R1*-Cas #3 plants. The 23-bp region is shaded, and the nucleotide sequence of the miR166 recognition site is shown in white letters. 5'-CTGG-3' sequences are boxed. Arrowheads show the inferred boundary sites of the 23-bp region, and the primers used to detect the *RDD1* transcripts with (#3 +23-bp primer) and without the 23-bp region (#3 −23-bp primer) are indicated by an arrow and interrupted arrow, respectively. (**b**) The inferred secondary structure of the 23-bp region in the *RDD1* transcript of the *R1*-Cas #3 plants. The nucleotide sequence of the 23-bp region is shaded, and that of the miR166 recognition site is denoted by white letters. 5'-CUGG-3' sequences are boxed, and G–U base pairs are shown by double dots. (**c**) RT-PCR amplification of *RDD1* in addition to synthetic miR166. Total RNAs from the shoots of the *R1*-Cas #3 plants at the 4-leaf stage were reverse-transcribed in the absence (−) or presence (+) of synthetic miR166 RNA to detect the RT-PCR products with (+23-bp) or without (−23-bp) the 23-bp region. Plants were grown in a low concentration hydroponic nutrient solution under normal conditions and harvested 2 h after the onset of illumination. The transcript levels are normalized to those of *RUBQ2*, and the highest transcript level is defined as 100. Data are presented as the average ± standard error of the mean (*n* = 3).

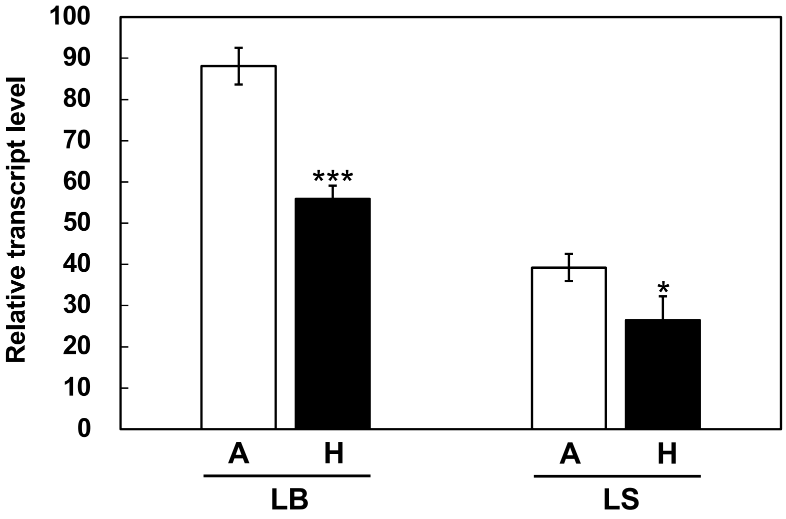

Supplementary Figure S3. *RDD1* transcript levels in the leaf blades (LB) and leaf sheaths (LS) of the wild-type plants at the 5-leaf stage. The plants were grown in soil in a growth chamber under ambient CO_2 (A) and high CO_2 conditions (1000 µmol CO_2 mol^{-1}) (H) and harvested 4 h after the onset of illumination. The transcript levels are normalized to those of *RUBQ2*, and the highest transcript level is defined as 100. Data are presented as the average ± standard error of the mean (n = 4). Asterisks indicate significant differences relative to the wild-type plants grown under ambient CO_2 conditions based on the Student's *t*-test (*$p < 0.05$ and ***$p < 0.001$).
